# Ambulatory intracranial pressure monitoring in NPH with a miniaturized acquisition chain

**DOI:** 10.1186/2045-8118-12-S1-P32

**Published:** 2015-09-18

**Authors:** Romain Manet, Laurent Gergele

**Affiliations:** 1 Department of Neurosurgery, University hospital of Saint-Etienne, France; 2 Department of Intensive Care, University hospital of Saint-Etienne, France

## Introduction

Diagnosis of cerebrospinal fluid (CSF) disorders may require intracranial pressure (ICP) monitoring. Classically, recordings are performed with a cumbersome acquisition chain, requiring the patient to be bedridden in a specific environment. New solutions have recently been proposed to free patient of these constraints, by mean of telemetric technology. We report our first experience of the use of a miniaturized ambulatory acquisition chain.

## Material and methods

Case report.

## Results

We report the case of a 78yrs patient, suspected of normal pressure hydrocephalus (NPH), with non-contributive infusion test. ICP has been recorded during 36hrs, combining miniaturized elements of a classical acquisition chain. Thanks to its compact size, the device allowed patient to be free to have normal activity during the recordings, in particular to stand and walk out of the ward. Like with telemetric systems, ambulatory ICP monitoring is more realistic than bed monitoring. In this first case, monitoring did not show rise of ICP at any moment or any position, eliminating diagnosis of NPH. However, recordings confirmed the negativity of ICP in standing position, as reported in other publications using telemetric systems.

## Conclusions

Recent publications concerning telemetric system of ICP recording are encouraging. However, reported morbidity (in particular infection rates) remains significantly higher than with classic ICP recording technics. In this reported case, we use a conventional ICP probe, with classical surgical technique. The large experience of intensive care confirmed the safety and the very low morbidity (in particular infectious risk) of classical ICP monitoring. Combining advantage of both methods (reduced bulk, low morbidity), miniaturized device could represent an interesting option for ambulatory ICP monitoring.

